# Association Analysis of Single Nucleotide Polymorphisms in the 5′ Regulatory Region of the IL-6 Gene with *Eimeria tenella* Resistance in Jinghai Yellow Chickens

**DOI:** 10.3390/genes10110890

**Published:** 2019-11-05

**Authors:** Hailiang Yu, Wenbin Zou, Shijie Xin, Xiaohui Wang, Changhao Mi, Guojun Dai, Tao Zhang, Genxi Zhang, Kaizhou Xie, Jinyu Wang, Cong Qiu

**Affiliations:** 1 College of Animal Science and Technology, Yangzhou University, Yangzhou 225009, China; hailiangyu122514@163.com (H.Y.); wenbinzou1216@163.com (W.Z.); shijiexin123@163.com (S.X.); wxh9409161412@126.com (X.W.); mch15861328120@163.com (C.M.); zhangt@yzu.edu.cn (T.Z.); zgx1588@126.com (G.Z.); yzxkz168@163.com (K.X.); jywang@yzu.edu.cn (J.W.); 2 Jiangsu Jinghai Poultry Group Co., Ltd., Haimen 226100, China; qiu008@sohu.com

**Keywords:** Jinghai yellow chicken, *IL-6* gene, *E. tenella*, single nucleotide polymorphisms (SNPs), haplotype

## Abstract

Interleukin 6 (*IL-6*) is an immunoregulatory cytokine involved in various inflammatory and immune responses. To investigate the effects of single nucleotide polymorphisms (SNPs) and haplotypes of *IL*-6 on resistance to *Eimeria tenella* (*E. tenella*), SNPs in the 5′ regulatory region of *IL*-6 were detected with direct sequencing, and the effects of SNPs and haplotypes on resistance to *E. tenella* were analyzed by the least square model in Jinghai yellow chickens. Nineteen SNPs were identified in the 5′ regulation region of *IL-6*, among which three SNPs were newly discovered. The SNP association analysis results showed that nine of the SNPs were significantly associated with *E. tenella* resistance indexes; the A-483G locus was significantly associated with the GSH-Px, IL-2, and IL-17 indexes (*p* < 0.05); the C-447G locus was significantly associated with the SOD, GSH-Px, IL-17, and IL-2 indexes (*p* < 0.05); and the G-357A locus had significant effects on the CAT and IL-16 indexes (*p* < 0.05). Haplotype analysis showed that H2H3 and H2H5 were favorable haplotype combinations with good coccidium resistance. Furthermore, we used qRT-PCR and observed that the expression of *IL-6* in the infection group was higher than that in the control group in the liver, proventriculus, small intestine, thymus, kidney, and bursa of Fabricius and extremely significantly different than that in the cecum especially (*p* < 0.01). In summary, SNPs and haplotypes in the 5′ regulatory region of *IL-6* have important effects on *E. tenella* resistance, and the results will provide a reference for molecular marker selection of *E. tenella* resistance in Jinghai yellow chickens.

## 1. Introduction

Coccidiosis is one of the intestinal parasitic diseases caused by the genus *Eimeria*, which seriously affects poultry production and animal welfare [1,2]. There are currently at least seven species of *Eimeria* found in chickens, among which the *E. tenella* parasite in the cecum is most common, mainly characterized by cecal epithelium hemorrhage and bloody stools [3,4]. So far, coccidiostats, live vaccines and strict hygiene control are the main measures to prevent coccidiosis in poultry production [5]. However, problems such as drug residues in food-producing animals, coccidiosis resistance, and vaccine safety still cannot be solved [6]. Therefore, studying the genes and SNPs associated with coccidia at the molecular level and breeding new strains resistant to coccidiosis are effective ways to solve this problem.

IL-6 is a pleiotropic cytokine that plays an important role in inflammation, the immune response, and hematopoiesis [7,8]. Produced mainly by macrophages, IL-6 performs protective functions in the healing of damaged tissues, participating in the acute phase immune response and coagulation in chickens [9]. Zhang and Zheng [10] reported that IL-6 triggers systemic inflammatory signals to initiate the host’s defense when the body is infected and damaged by pathogens. Rose-John et al. [11] discovered that the host’s defense against bacterial and fungal pathogen infection relied mainly on the classical IL-6 signaling pathway. By studying the effect of DNA plasmids carrying chicken *IL-6* on infectious bursal disease virus (IBDV), Sun et al. [12] determined that injection with IL-6 plasmids resulted in a significantly increased protective effect in chickens. The RNA-seq technique was used to sequence the cecum tissue of an *E. tenella*-infected group and a noninfected group on the 7th day post-infection, and the results revealed that the differentially expressed genes (DEGs) included *IL-6*, *IL-12β*, and *TGFB2*, which were significantly enriched during coccidiosis infection [13]. In addition, Zhang et al. [14] showed that *IL-1β*, *IL-6*, and *TNF-α* play vital roles in the inflammatory response of chicken embryo fibroblasts infected with avian reovirus. However, there are relatively few studies on the association between single nucleotide polymorphisms (SNPs) of the chicken *IL-6* gene and coccidium resistance. We previously performed a bioinformatics analysis of *IL-6* gene SNPs in the 5′ promoter region of the Jinghai yellow chicken. In the present study, DNA direct sequencing technology was used to analyze the effect of SNPs and haplotypes in the 5′ regulatory region of *IL-6* gene on *E. tenella* resistance indexes, and the results will provide basic data for molecular marker selection breeding of *E. tenella* resistance in the Jinghai yellow chicken.

## 2. Materials and Methods

### 2.1. Animals

A total of 220 (110 ♂, 110 ♀) 1-day-old healthy and physiologically similar Jinghai yellow chickens were randomly selected from the Jinghai Yellow Chicken Resource Farm (Haimen, China), housed in a sterile animal room, and fed an antibiotic-free diet until 30 days old. All the chickens were free from parasitic infection according to fecal detection during the pre-test period. Each chicken in the infected group (110 birds—5M + 55F) was given an oral infection with 2.5 × 10^4^ sporulated oocysts, and chickens in the uninfected group (110 birds—55M + 55F), receiving no oocysts, were used as uninfected controls. Parasite oocysts were collected from the Department of Parasitology, College of Veterinary Medicine, Yangzhou University [15]. The experiments were carried out in strict accordance with the regulation of the Administration of Affairs Concerning Experimental Animals (Ministry of Science and Technology, Yangzhou, China, revised in June 2012) and approved by experimental animal use permit No. SYXK (Su) 2012-0029.

### 2.2. Genomic DNA Extraction

Blood samples were obtained from the wing vein of each bird on the 7th day post-infection, and the blood was anticoagulated by heparin sodium. After centrifugation, blood cells and plasma were stored at −20 °C. Genomic DNA was extracted and purified using the conventional phenol-chloroform extraction method. The concentration of the extracted DNA samples was measured by an Eppendorf BioPhotometer (Eppendorf Scientific, Hamburg, Germany), and quality was based on gel electrophoresis.

### 2.3. Resistance Index Detection

The activity of CAT, SOD, and GSH-PX and the concentrations of NO, MDA, IL-2, IL-16, IL-17, IFN-γ, and β-carotene in plasma on the 7th day post-infection were determined by ELISA according to the manufacturer’s instructions (Nanjing Jiancheng Bioengineering Research Institute, Nanjing, China).

### 2.4. Primer Design and Polymerase Chain Reaction (PCR) Conditions

Based on the GenBank (https://www.ncbi.nlm.nih.gov/genbank/) chicken genomic DNA sequence (accession number: NC_006089.4), four pairs of primers were designed using Primer Premier 5.0 (PREMIER Biosoft, Palo Alto, CA, USA). The amplified fragments were located between −2200 bp and −1 bp upstream of the *IL-6* gene. The primers were synthesized by Sangon Biotech Co. (Shanghai, China). Primer sequences can be seen in Table 1.

PCR was carried out in a reaction containing 1 μL of DNA template, 10 μL of 2× Taq Master Mix, 1 μL each of the upstream and downstream primers, and by adding ddH_2_O to 20 μL. The PCR amplification procedures were preliminary denaturation at 95 °C for 5 min; 35 cycles of denaturation at 95 °C for 30 s, annealing for 30 s at the optimum primer annealing temperature, and elongation for 1 min at 72 °C; and a final extension at 72 °C for 7 min. The samples were stored at 4 °C.

### 2.5. SNP Identification in 5′ Regulatory Region of the IL-6 Gene

The purified PCR products were sent to Sangon Biotech Co. (Shanghai, China) and sequenced using an ABI3730XL DNA analyzer (Applied Biosystems, Foster City, CA, USA). We compared the sequences with the reference on NCBI-BLAST (https://blast.ncbi.nlm.nih.gov/Blast.cgi) to discover potential SNPs of the chicken *IL-6* gene by DNAMAN 5.2 (Lynnon Corporation, San Ramon, CA, USA) and MEGA6.06.

### 2.6. Haplotype Analysis and Estimation of Linkage Disequilibrium (LD)

SHEsis analysis software (http://analysis.bio-x.cn/SHEsisMain.htm) was used to estimate the extent of LD between the identified SNPs, and an imbalance coefficient of D’ > 0.8 with a correlation coefficient of r^2^ > 0.33 is considered a strong LD between the SNPs [16]. Furthermore, we used PHASE2.1 to calculate the type of haplotype and its frequency.

### 2.7. Statistical Analysis

Popgene 1.32 software was employed to analyze the population genetic diversity of identified SNPs. The associations of the SNPs and haplotype with the *E. tenella* resistance index in Jinghai yellow chickens were analyzed by the least square model:

Y = μ + Sex + Group + Genotype + (Group × Genotype) + e,

where *Y* is the measured value of the resistance index for each bird; *μ* is the overall mean; *Sex* is the effect of sex; *Group* is the effect of the infected and uninfected groups; *Genotype* is the genotype or haplotype combination effect; *Group × Genotype* is the interaction effects between different infection states and genotypes; and *e* is the random error effect.

All statistical analyses were performed using the univariate model of the general linear model (GLM) in SPSS 25.0 statistical software, and multiple comparisons were performed using the least significant difference (LSD) method.

### 2.8. Quantitative Real-Time PCR (qRT-PCR)

We randomly selected ten chickens for qRT-PCR from each group of the infected and control. qRT-PCR primers for *IL-6* were designed using Primer-BLAST on the NCBI website (F: TGGTGATAAATCCCGATGAAG, R: GGCACTGAAACTCCTGGTCT). GAPDH and β-actin were used as reference genes [17]. qRT-PCR was performed using an ABI Prism 7500 sequence-detection system (Applied Biosystems, Foster City, CA, USA) with a SYBR Green PCR Master Mix (TaKaRa, Dalian, China) according to the manufacturers’ instructions. The procedure was as follows: preliminary denaturation at 94 °C for 30 s, followed by 40 cycles of denaturation for 5 s at 95 °C and annealing for 34 s at 60 °C. The individual measurements were performed in triplicate, and the relative gene expression was calculated using the 2^−ΔΔ*C*t^ method.

## 3. Results

### 3.1. SNP Identification in IL-6

By sequencing and sequence alignment, a total of nineteen SNP sites were identified in the 5′ regulation region of *IL-6* and three genotypes were detected in each site, among which three SNPs were newly discovered (C-447G, G-357A, A-663G) [18]. Based on the polymorphism information analysis, the G-865A, A-663G and A-400G loci were lowly polymorphic (PIC < 0.25), and sixteen SNP sites were moderately polymorphic (0.25 < PIC < 0.5). The χ^2^-test indicated that fourteen SNP sites were consistent with the Hardy–Weinberg equilibrium. The chromosome position and variation of all the identified SNPs are summarized in Table 2.

### 3.2. Association between SNPs and the E. tenella Resistance Index

Associations between SNPs of the *IL-6* gene and *E. tenella* resistance indexes are presented in Table 3. We found that these nine SNPs were significantly associated with at least one coccidiosis resistance index. At the T-1534C locus, the MDA concentration of the CT genotype was significantly higher than that of the CC genotype (*p* < 0.01) and significantly higher than that of the TT genotype (*p* < 0.05), but there was no significant difference among the three genotypes for other resistance indexes (*p* > 0.05). The MDA concentration of CG genotypes at the C-939G locus was significantly higher than that of the CC genotype (*p* < 0.01) and significantly higher than that of the GG genotype (*p* < 0.05). At the G-889A locus, the IL-2 level of the GA genotype was significantly higher than that of the AA genotype (*p* < 0.01), and those of the GA genotype were also higher than those of the other two genotypes for the NO, CAT, IL-17, IFN-γ, and weight gain indexes, but the difference was not significant (*p* > 0.05).The IL-2 indexes of AG genotypes at A-634G locus were significantly higher than that of AA and GG genotypes (*p* < 0.05) but significantly lower than those of GG genotypes for GSH-Px indexes. At the C-511T locus, the NO concentration of the TT genotype was significantly higher than that of the CC genotype (*p* < 0.05). At the A-483G locus, the GSH-Px, IL-17, and IL-2 indexes of the GG genotype were significantly higher than those of the GA and AA genotype (*p* < 0.05), and those of the GG genotype were also higher than those of the other two genotypes for the NO, CAT, SOD, IL-16, and weight gain indexes, but the difference was not significant (*p* > 0.05). The IL-2 level of the GT genotype at T-458G locus was significantly higher than that of the TT genotype (*p* < 0.05) and significantly higher than that of the GG genotype (*p* < 0.01). The SOD, IL-17, GSH-Px, and IL-2 indexes of CC genotypes at the C-447G locus were significantly different from those of the other two genotypes (*p* < 0.05). At the G-357A locus, the CAT and IL-16 indexes of the AA genotype were significantly higher than those of the GG and GA genotypes (*p* < 0.05) but significantly lower than that of the GG and GA genotypes (*p* < 0.01) for the IL-17 level.

### 3.3. Association between Haplotypes and the E. tenella Resistance Indexes

**Figure 1:**
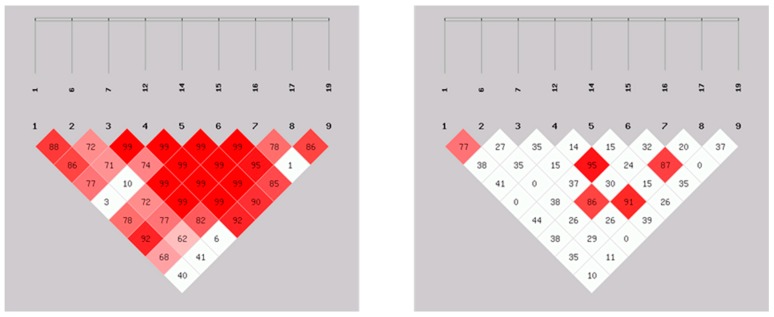
D’ values (**left**) and r^2^ values (**right**) of pairwise LD analysis of the *IL-6* gene. Note: the larger the value, the darker the color in boxes, the stronger the LD.

SHEsis software LD analysis was employed to estimate the extent of LD between the nine identified SNPs; the results are presented in Figure 1. The deeper the red color is in the figure, the stronger the LD. D’ greater than 0.8 shows a distinct red color, and the correlation coefficient r^2^ is greater than 0.33, which indicates a strong LD. The results showed that there was a strong LD between the G-889A and T-458G loci and between the A-634G and A-483G loci, and the A-634G, A-483G and C-447G loci were also highly linked.

Moreover, haplotype analysis showed that ten haplotypes were formed by the nine SNPs, consisting of H1, H2, H3, H4, H5, etc. and that the frequency of the haplotypes was greater than 1% (Table 4). Haplotype-based association analysis showed that the resistance indexes for IL-17, GSH-Px, MDA, IL-2, IL-16, and weight gain of the H2H3 and H2H5 haplotype combinations were significantly (*p* < 0.05) or extremely significantly (*p* < 0.01) higher than those of the other haplotype combinations. IFN-γ and SOD indexes of the H2H3 and H2H5 genotypes were also higher than those of the other haplotypes, but the difference was not significant (*p* > 0.05). The information is presented in Table 5.

### 3.4. Expression of the IL-6 Gene in Nine Tissues

**Figure 2:**
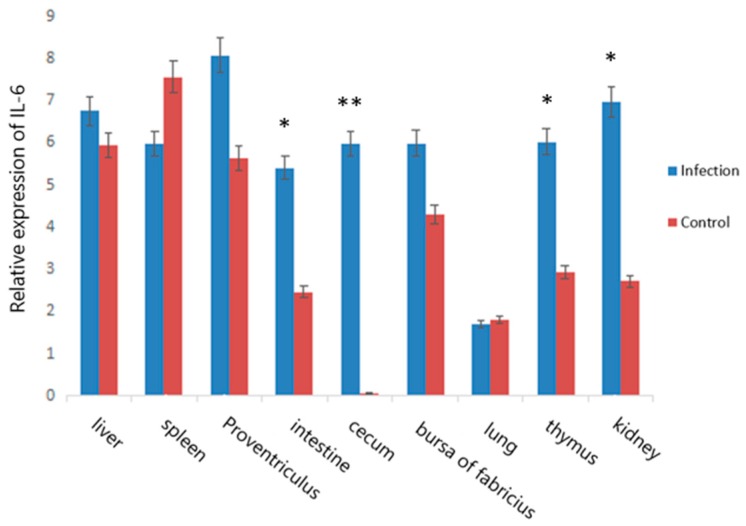
Relative expression of the *IL-6* gene in nine tissues of Jinghai yellow chickens. Note: “*” indicates a significant difference (*p* < 0.05), “**” indicates that the difference is extremely significant (*p* < 0.01).

By using qRT-PCR, a comparison of *IL-6* gene expression among different tissues was performed, and the results are presented in Figure 2. We found that the *IL-6* gene was expressed in all nine tissues. The expression of *IL-6* in the infection groups was higher than that in the control groups in the liver, proventriculus, small intestine, thymus, kidney, and bursa of Fabricius of Jinghai yellow chickens, and the expression level in the cecum was significantly higher in the infected group than in the control group (*p* < 0.01).

## 4. Discussion

Research on the function and clinical application of the chicken *IL-6* gene is still being conducted. Studies have shown that chicken *IL-6* exhibits significant potential as an immune adjuvant, immune enhancer, and antibiotic substitute [19,20,21]. Rose-John et al. [11] discovered that the host’s defense against bacterial and fungal pathogen infection relied mainly on classical *IL-6* signaling pathways. Swaggerty et al. [22] reported that high levels of *IL-6*, *CXCLi2* (*IL-8*), and the chemokine *CCLi2* expressed in broiler chickens resulted in significantly higher resistance than in those with low expression. These studies provide a basis for studying the relationship between *IL-6* and coccidiosis infection. In this study, we used DNA direct sequencing technology to detect SNPs in the *IL-6* gene 5′regulation region of the Jinghai yellow chicken and identified nineteen SNPs. Among them, three SNPs were newly discovered. All of the identified SNPs were lowly or moderately polymorphic, indicating that the genetic diversity among individuals within the population was good, which is conducive to subsequent breeding.

By bioinformatics analysis of the polymorphism of the promoter region of the *IL-6* gene, Xin et al. [18] found that eleven SNPs are located in the core promoter region and may influence *IL-6* gene expression by altering transcription factor binding or CpG methylation status. Pang et al. [23] analyzed the correlation between the SNPs in the *IL-6* gene promoter region and the severity of enterovirus EV71 infection, indicating that *IL-6* has a potential correlation with severe infection of human EV71. Zhang et al. [24] reported that SNPs in the *MHC B-F* and *SPOCK1* genes are significantly associated with resistance to *Salmonella pullorum*. In this study, we analyzed the association between SNPs of the *IL-6* gene in the regulation region and coccidium resistance indexes and found that nine SNPs were significantly associated with *E. tenella* resistance. The GG genotype of the A-483G locus was significantly higher than the GA and AA genotypes in the GSH-Px, IL-17, and IL-2 indexes. The NO, CAT, SOD, IL-16, and weight gain indexes of the GG genotype were also higher than those of the other two genotypes. Regarding the newly discovered SNPs, the CC genotype of the C-447G locus was significantly higher than the GG and CG genotypes in the SOD, GSH-Px, IL-17, and IL-2 indexes and higher than the GG and CG genotypes in the NO index. The AA genotype of the G-357A locus was significantly higher than the GG and GA genotypes in the CAT and IL-16 indexes and higher than the GG and GA genotypes in the SOD, MDA, β-carotene, and weight gain indexes. These resistance-related mutation sites may be involved in the regulation of *IL-6* gene expression and affect its immune function during *Eimeria* infection.

SNPs on the same chromosome do not exist alone, and adjacent-allele SNPs may work simultaneously, indicating LD [25]. The use of haplotypes in association studies to identify common variants may be more effective than single allele studies [26,27]. Most traits are regulated by multiple genes or multiple sites of a certain gene, and it is impossible to accurately determine the true correlation between genes and traits by analyzing the polymorphism of a single locus in a certain gene. In this study, the haplotype combination association analysis results showed that the H2H3 and H2H5 haplotype combinations were significantly higher than other haplotype combinations in the resistance indexes for IL-17, GSH-Px, MDA, IL-2, IL-16, and weight gain. Thus, the H2H3 and H2H5 haplotype combinations are the dominant haplotype combinations of chickens against *Eimeria* infection.

Furthermore, we used qRT-PCR to reveal that the *IL-6* gene was expressed in all nine tissues. The expression of *IL-6* in the infection groups was higher than that in the control groups in the liver, proventriculus, small intestine, thymus, kidney, and bursa of Fabricius and significantly higher than that in the control group in the cecum. *E. tenella* is one of the most pathogenic species that exclusively occupies the cecum, causing significant swelling and bleeding of the cecum [28,29]. The *Eimeria* species infect cecum epithelial cells, with the potential to fully occupy the infection sites without appropriate control measurements [30]. Therefore, the study data suggest that *IL-6* may play an important role in *E. tenella* resistance in Jinghai yellow chickens.

## 5. Conclusions

In this study, nineteen SNPs were identified in the 5′ regulation region of *IL-6*, among which nine of the SNPs were significantly associated with *E. tenella* resistance indexes, The A-483G locus were significantly associated with GSH-Px, IL-2, and IL-17 indexes; the C-447G locus were significantly associated with SOD, GSH-Px, IL-17, and IL-2 indexes; the G-357A locus had significant effects on the CAT and IL-16 indexes. Haplotype analysis showed that H2H3 and H2H5 were the favorable haplotype combinations with good coccidia resistance. Our results will provide a reference for molecular marker selection of *E. tenella* resistance in Jinghai Yellow Chicken.

## Figures and Tables

**Table 1:** Primer sequences for PCR amplification of the chicken *IL-6* gene.

Primer	Primer Sequence (5′-3′)	Length/bp	Annealing Temperature/°C
P1	F: AGAGAGGACTAACCCACAGAG R: CCAGCTTCTCCAGTCTTGTC	698	58.5
P2	F: AGGGACAGCAATGGCAGAAG R: AAGAGCTGATCCTGGTTCTGG	717	60.5
P3	F: CAGAGGACGTCCTACCTCAA R: GGTGAGCCTGGCAGCC	690	56.5
P4	F: AAGATAAGACGCGCCACACC R: TTGAGGTTGTTCCGGACGAG	803	58.5

**Table 2:** Information on single nucleotide polymorphisms (SNPs) identified in the *IL-6* gene.

NO	SNP Name	Position	Genotype	Genotype Frequency	Allelic Gene	Allele Frequency	χ^2^ Value	*p* Value	H	Ne	PIC
1	T-1534C	30947851	CC	0.400	C	0.618	0.123	0.726	0.472	1.894	0.361
			TT	0.164	T	0.382					
			CT	0.436							
2	C-1280T	30948105	CC	0.636	C	0.818	1.604	0.205	0.298	1.424	0.253
			TT	0	T	0.182					
			CT	0.364							
3	G-1223A	30948162	GG	0.582	G	0.791	2.636	0.104	0.331	1.494	0.276
			AA	0	A	0.209					
			AG	0.418							
4	G-1220T	30948165	GG	0.600	G	0.800	2.260	0.133	0.320	1.471	0.269
			TT	0	T	0.200					
			GT	0.400							
5	A-1208G	30948177	AA	0.600	G	0.800	2.260	0.133	0.320	1.471	0.269
			GG	0	T	0.200					
			AG	0.400							
6	C-939G	30948446	CC	0.418	C	0.618	0.840	0.359	0.472	1.894	0.361
			GG	0.182	G	0.382					
			CG	0.400							
7	G-889A	30948496	GG	0.254	G	0.545	1.173	0.279	0.496	1.984	0.361
			AA	0.164	A	0.455					
			AG	0.582							
8	G-865A	30948520	AA	1	A	1			0.000	1.000	0.000
			GG	0	G	0					
			AG	0							
9	G-791C	30948594	GG	0.218	G	0.518	1.675	0.196	0.499	1.998	0.375
			CC	0.182	C	0.482					
			GC	0.600							
10	G-734A	30948651	GG	0.473	G	0.682	0.007	0.933	0.434	1.766	0.400
			AA	0.109	A	0.318					
			GA	0.418							
11	A-663G	30948722	GG	1	G	1			0.000	1.000	0.000
			AA	0	A	0					
			GA	0							
12	A-634G	30948751	GG	0.473	G	0.700	0.133	0.715	0.420	1.724	0.332
			AA	0.073	A	0.300					
			GA	0.454							
13	G-610A	30948775	GG	0.527	G	0.764	3.966	0.046	0.361	1.565	0.296
			AA	0.000	A	0.236					
			GA	0.473							
14	C-511T	30948874	TT	0.545	T	0.745	0.020	0.888	0.380	1.612	0.307
			CC	0.055	C	0.255					
			TC	0.400							
15	A-483G	30948902	AA	0.454	A	0.691	0.302	0.583	0.427	1.746	0.336
			GG	0.073	G	0.309					
			AG	0.473							
16	T-458G	30948927	TT	0.327	T	0.582	0.022	0.882	0.487	1.948	0.368
			GG	0.164	G	0.418					
			TG	0.509							
17	C-447G	30948938	CC	0.455	C	0.682	0.018	0.893	0.434	1.766	0.400
			GG	0.091	G	0.318					
			CG	0.454							
18	A-400G	30948985	GG	0.964	G	0.982	12.882	0.000	0.036	1.037	0.035
			AA	0	A	0.018					
			AG	0.036							
19	G-357A	30949028	GG	0.291	G	0.518	0.219	0.640	0.499	1.997	0.375
			AA	0.254	A	0.482					
			AG	0.455							

Note: PIC: Polymorphism information content (PIC > 0.5 indicates highly polymorphic; 0.25 < PIC < 0.5 indicates moderately polymorphic; PIC < 0.25 indicates lowly polymorphic); H: Heterozygosity; Ne: Effective number of alleles.

**Table 3:** Associations of nine SNPs in the *IL-6* gene with the *E. tenella* resistance indexes in chickens.

Mutation Site	Genotype	NO (μmol/L)	CAT (U/L)	SOD (U/L)	IL-17 (pg/mL)	GSH-Px (U/L)	MDA (mmol/L)	IL-2 (ng/L)	IL-16 (ng/L)	IFN-γ (ng/L)	β-Carotene (μmol/L)	Weight Gain (g)
T-1534C	CC	55.92 ± 5.17	66.25 ± 10.54	126.23 ± 14.42	37.36 ± 5.41	482.33 ± 83.44	5.70 ± 0.76 ^Bb^	29.71 ± 3.34	52.15 ± 7.60	37.19 ± 6.02	63.40 ± 26.13	33.09 ± 26.14
	TT	52.33 ± 5.73	63.54 ± 14.61	117.56 ± 29.92	35.56 ± 9.78	449.50 ± 60.79	5.10 ± 0.94 ^ABb^	33.27 ± 5.70	55.34 ± 11.23	34.65 ± 2.55	67.42 ± 16.90	43.73 ± 15.79
	CT	54.18 ± 5.40	61.83 ± 10.03	120.44 ± 19.71	40.23 ± 8.65	443.38 ± 66.52	5.98 ± 0.79 ^Aa^	34.57 ± 6.06	53.04 ± 7.15	35.62 ± 4.08	66.40 ± 29.01	69.55 ± 114.34
C-939G	CC	56.99 ± 5.22	65.82 ± 11.00	126.14 ± 14.55	37.14 ± 4.97	509.62 ± 88.23	5.72 ± 0.77 ^Bb^	30.17 ± 3.85	51.28 ± 8.62	37.16 ± 6.05	60.55 ± 23.18	36.75 ± 22.49
	GG	52.20 ± 4.88	61.96 ± 12.70	116.14 ± 25.65	36.69 ± 9.46	434.06 ± 60.67	5.20 ± 0.85 ^ABb^	31.61 ± 6.18	54.40 ± 9.85	34.48 ± 2.22	73.80 ± 19.00	36.84 ± 22.32
	CG	53.83 ± 5.32	62.53 ± 10.27	121.35 ± 20.36	40.37 ± 8.83	433.93 ± 47.83	6.02 ± 0.79 ^Aa^	35.12 ± 5.67	53.66 ± 6.65	35.81 ± 4.23	65.31 ± 30.16	72.88 ± 119.70
G-889A	GG	53.39 ± 5.08	59.09 ± 12.07	126.83 ± 28.18	36.55 ± 6.79	431.79 ± 45.54	5.68 ± 1.24	31.31 ± 4.75 ^ABa^	55.81 ± 7.35	35.09 ± 2.22	58.64 ± 17.03	48.09 ± 18.03
	AA	54.56 ± 3.31	63.23 ± 9.43	118.92 ± 8.42	37.68 ± 6.55	482.46 ± 78.25	5.99 ± 0.68	27.76 ± 3.19 ^Bb^	53.54 ± 4.23	35.01 ± 3.55	76.44 ± 31.73	34.00 ± 26.98
	GA	54.77 ± 5.93	65.14 ± 10.45	120.03 ± 17.82	39.81 ± 8.86	459.45 ± 77.90	5.75 ± 0.68	34.79 ± 5.64 ^Aa^	51.94 ± 8.49	36.45 ± 5.36	66.42 ± 28.19	62.73 ± 111.00
A-634G	GG	54.93 ± 5.56	62.66 ± 10.95	114.96 ± 16.61	40.55 ± 6.25	462.62 ± 82.99 ^a^	5.38 ± 0.99	31.87 ± 4.90 ^B^	49.35 ± 9.44	36.38 ± 4.84	67.48 ± 22.40	40.36 ± 22.27
	AA	55.31 ± 4.81	68.05 ± 9.15	131.36 ± 32.52	33.34 ± 7.28	386.79 ± 85.77 ^b^	5.33 ± 1.01	30.76 ± 4.71 ^AB^	54.13 ± 10.38	35.92 ± 4.24	54.08 ± 16.31	55.53 ± 7.66
	AG	54.38 ± 5.64	57.48 ± 11.77	117.56 ± 20.52	38.97 ± 7.91	420.91 ± 45.95^b^	5.70 ± 0.81	35.95 ± 5.73 ^A^	53.56 ± 8.41	37.32 ± 5.04	62.50 ± 30.51	68.07 ± 106.66
C-511T	TT	55.68 ± 5.19 ^a^	61.54 ± 11.50	120.39 ± 19.95	38.34 ± 7.63	424.69 ± 63.35	5.53 ± 0.80	33.64 ± 6.15	51.97 ± 8.36	36.64 ± 5.09	64.95 ± 32.25	65.25 ± 97.93
	CC	49.13 ± 5.34 ^b^	56.24 ± 7.86	100.24 ± 8.98	45.90 ± 3.36	425.87 ± 52.67	4.77 ± 0.90	35.57 ± 1.55	48.27 ± 13.37	37.97 ± 2.03	80.04 ± 18.71	67.43 ± 11.22
	CT	54.14 ± 5.54 ^ab^	60.14 ± 12.15	115.49 ± 19.73	39.72 ± 6.73	458.16 ± 7.23	5.60 ± 1.04	33.39 ± 5.29	51.57 ± 9.95	36.78 ± 4.87	61.12 ± 14.81	36.97 ± 17.39
A-483G	GG	55.31 ± 4.81	68.05 ± 9.15	131.36 ± 32.52	39.98 ± 5.65 ^a^	462.00 ± 84.63 ^a^	5.33 ± 1.01	36.24 ± 5.80 ^A^	54.13 ± 10.38	35.92 ± 4.24	54.08 ± 16.31	55.53 ± 7.66
	AA	54.85 ± 5.66	62.06 ± 10.73	113.67 ± 15.58	31.34 ± 7.28 ^b^	386.79 ± 85.77 ^b^	5.33 ± 0.98	31.41 ± 4.40 ^B^	49.59 ± 9.55	36.25 ± 4.90	67.41 ± 22.85	40.59 ± 22.70
	GA	54.49 ± 5.56	58.26 ± 12.19	118.70 ± 20.93	39.57 ± 8.34 ^a^	423.11 ± 46.41 ^b^	5.74 ± 0.81	30.76 ± 4.71 ^AB^	53.16 ± 8.48	37.40 ± 4.95	62.76 ± 29.93	46.93 ± 16.87
T-458G	TT	52.86 ± 5.41	59.55 ± 11.43	120.15 ± 25.74	40.42 ± 7.84	428.76 ± 54.92	5.51 ± 1.05	33.67 ± 5.56 ^ABb^	53.89 ± 8.51	36.49 ± 3.05	63.42 ± 17.26	50.26 ± 17.31
	GG	55.33 ± 5.47	62.75 ± 11.46	115.91 ± 8.96	39.59 ± 5.34	460.75 ± 66.46	5.28 ± 1.08	28.94 ± 4.49 ^Bb^	50.62 ± 6.55	34.48 ± 3.32	73.66 ± 31.80	39.59 ± 21.75
	GT	55.70 ± 5.38	60.78 ± 11.88	115.98 ± 18.26	38.50 ± 7.47	436.91 ± 82.92	5.60 ± 0.77	35.14 ± 5.28 ^Aa^	50.47 ± 10.16	37.69 ± 5.92	61.74 ± 28.74	42.73 ± 20.08
C-447G	CC	55.00 ± 5.66	62.99 ± 11.05	131.24 ± 28.28 ^a^	40.76 ± 6.29 ^a^	461.06 ± 84.30 ^a^	5.36 ± 1.01	35.06 ± 4.90 ^a^	49.31 ± 9.63	36.20 ± 4.86	65.59 ± 20.63	40.52 ± 22.71
	GG	54.86 ± 4.29	65.32 ± 10.01	110.53 ± 16.81 ^b^	33.73 ± 6.37 ^b^	409.79 ± 90.35 ^ab^	5.40 ± 0.89	30.03 ± 4.40 ^b^	53.38 ± 9.15	36.89 ± 4.27	66.21 ± 30.57	51.66 ± 10.90
	GC	54.38 ± 5.64	57.49 ± 11.76	117.56 ± 20.52 ^ab^	38.97 ± 7.91 ^ab^	420.91 ± 45.95 ^b^	5.70 ± 0.81	32.95 ± 5.73 ^b^	53.56 ± 8.41	37.32 ± 5.04	62.50 ± 30.51	47.43 ± 17.02
G-357A	GG	55.17 ± 5.84	62.64 ± 11.23 ^ab^	115.36 ± 20.47	43.01 ± 8.87 ^A^	457.92 ± 97.76	5.42 ± 1.00	35.07 ± 4.54	47.75 ± 10.78 ^b^	37.77 ± 5.25	63.50 ± 16.30	41.09 ± 24.06
	AA	54.58 ± 5.25	65.27 ± 11.70 ^a^	123.06 ± 21.04	35.71 ± 5.85 ^B^	425.97 ± 68.38	5.63 ± 0.73	33.96 ± 5.18	55.39 ± 9.02 ^a^	35.61 ± 4.93	64.75 ± 35.90	50.62 ± 15.09
	GA	54.48 ± 5.54	56.90 ± 10.72 ^b^	115.40 ± 18.77	38.95 ± 5.76 ^AB^	432.31 ± 52.09	5.52 ± 0.97	32.56 ± 6.40	51.96 ± 7.24 ^ab^	36.78 ± 4.56	64.43 ± 25.57	43.64 ± 18.60

Note: ^a, b^ within the same column with different superscripts indicate a *p*-value < 0.05; ^A, B^ within the same column with different superscripts indicate a *p*-value < 0.01.

**Table 4:** Haplotype analysis for the 5′ untranslated region (UTR).

NO	Haplotype	Frequency
H1	TGGATGTGA	0.242
H2	CCAGTAGCG	0.226
H3	CCGGCATCG	0.145
H4	CCAGTAGCA	0.144
H5	TGGGCATCG	0.054
H6	CCGATGTGA	0.039
H7	TCGGCATCG	0.019
H8	TGAGCATCG	0.018
H9	TGAGTAGCA	0.011
H10	TGGATGTGG	0.010

**Table 5:** Associations of haplotype with the *E. tenella* resistance indexes in chickens.

Resistant Parameters	Haplotype Combination					
	H1H1	H1H2	H1H4	H1H5	H2H3	H2H5
NO (μmol/L)	55.62 ± 4.23	61.38 ± 5.01	58.38 ± 4.78	56.14 ± 4.77	56.89 ± 5.26	57.89 ± 4.92
CAT (U/L)	58.37 ± 9.10	62.77 ± 10.11	58.18 ± 9.98	60.18 ± 11.23	59.23 ± 9.11	61.23 ± 10.53
SOD (U/L)	117.11 ± 18.30	118.67 ± 18.67	118.29 ± 19.45	119.87 ± 19.29	121.59 ± 19.34	122.87 ± 18.99
IL-17 (pg/mL)	39.34 ± 6.10 ^a^	42.78 ± 5.78 ^a^	42.44 ± 6.23 ^a^	41.07 ± 6.19 ^a^	48.67 ± 6.34 ^b^	47.61 ± 5.99 ^b^
GSH-Px (U/L)	339.76 ± 68.63 ^A^	329.58 ± 70.54 ^A^	433.27 ± 67.90 ^B^	340.67 ± 67.78 ^A^	429.28 ± 67.33 ^B^	450.34 ± 71.37 ^B^
MDA (mmol/L)	5.02 ± 0.66 ^a^	5.44 ± 0.72 ^a^	6.01 ± 0.87 ^b^	5.32 ± 0.91 ^a^	5.92 ± 0.68 ^b^	5.99 ± 0.93 ^b^
IL-2 (ng/L)	30.36 ± 4.98 ^a^	31.67 ± 5.37 ^a^	35.11 ± 5.66 ^ab^	33.67 ± 5.43 ^a^	35.74 ± 5.17 ^ab^	39.04 ± 5.10 ^b^
IL-16 (ng/L)	50.13 ± 8.37 ^a^	50.38 ± 9.26 ^a^	50.88 ± 9.35 ^b^	50.56 ± 8.99 ^a^	52.11 ± 8.63 ^ab^	54.19 ± 9.19 ^b^
IFN-γ (ng/L)	35.81 ± 4.62	35.82 ± 5.29	36.14 ± 4.84	33.13 ± 4.02	37.09 ± 5.10	36.37 ± 4.61
β-carotene (μmol/L)	58.97 ± 19.87	61.13 ± 23.42	60.53 ± 24.98	63.47 ± 24.43	59.01 ± 23.77	58.16 ± 25.83
weight gain (g)	57.70 ± 44.11 ^a^	60.13 ± 57.24 ^a^	58.92 ± 49.78 ^a^	59.54 ± 50.34 ^a^	63.32 ± 47.92 ^b^	62.86 ± 56.98 ^b^

Note: ^a, b^ within the same line with different superscripts indicate a *p*-value < 0.05; ^A, B^ within the same line with different superscripts indicate a *p*-value < 0.01.
